# Practical effects of carbon emissions trading system on energy efficiency

**DOI:** 10.1038/s41598-023-50621-3

**Published:** 2024-01-02

**Authors:** Xue Li, Aochen Cao, Yuhan Zhang, Yuting Zhao, Lulu Chen, Pan Jiang, Liang Liu

**Affiliations:** 1 School of Economics and Management, Southwest University of Science and Technology, Mianyang, 621010 China; 2 School of Environment and Resources, Southwest University of Science and Technology, Mianyang, 621010 China; 3 School of Economics, Central University of Finance and Economics, Beijing, 102206 China

**Keywords:** Energy and society, Environmental economics, Environmental impact

## Abstract

The carbon emissions trading system (CETS) is a helpful policy instrument for separating carbon emissions from economic expansion, and it significantly impacts energy efficiency (EE). This study uses 30 Chinese provinces from 2007 to 2020 as its research samples, and classifies energy efficiency into single-factor energy efficiency (SFE) and total-factor energy efficiency (TFE), using the difference-in-differences model to examine the effect and mechanism of the CETS on EE. As an additional tool to assess the efficacy of the CETS, the corresponding evolution of the rebound effect of energy-related carbon emissions (RECE) is also calculated. This study shows that the CETS can significantly improve EE in China's pilot provinces. The influence mechanism indicates that the effect of the CETS on EE is influenced by the level of government governance, green innovation, and industrial structure optimization. Further study finds that after the CETS was carried out, the RECE in pilot provinces was higher than that in non-pilot provinces, and 31.4% of carbon emissions reduced by EE improvement rebounded. Therefore, the CETS has yet to realize its full carbon reduction potential. The study offers specific policy proposals for the enhancement of China's CETS in light of the aforementioned findings.

## Introduction

Global carbon dioxide emissions connected to energy reached a new high of more than 36.8 Gt in 2022, according to the *CO*_*2*_* Emissions in 2022* published by the International Energy Agency. Among them, China accounts for 1/3 of the total emissions and is still the world's largest carbon-emitting country. China still has a way to go before realizing the separation of carbon from energy. The Chinese government recommended in its report for 2022 encouraging the promotion of the dual control of energy consumption and intensity to the overall quantity and intensity of carbon emissions^1^. This is a major policy adjustment and shift after the energy intensity control target proposed in 2006 during China's 11th Five-Year Plan. Energy, as one of the indispensable factors of production, can also be decoupled from carbon emissions by adjusting the energy structure, improving the efficiency of energy use, or developing cleaner production technologies, rather than just emphasizing the reduction of total energy use^2^. Developed countries that have peaked in carbon emissions are gradually showing a trend of carbon and energy separation^3^, which indicates that energy use and carbon emissions are not in a single linear relationship. Energy efficiency (EE) improvement has become one of the major issues to be tackled to decouple energy use from carbon emissions in China. For the sake of reducing carbon emissions, China actively advocates a parallel policy and market strategy to enhance EE, and the carbon emission market trading system (CETS) has become a crucial tool for conserving energy and reducing the total amount and intensity of carbon emissions^4^. As a market-encouraging environmental regulation and energy efficiency improvement policy, its ability to reduce carbon emissions through improving EE is of great research value for achieving clean and efficient energy use and high-quality economic development.

**Figure 1:**
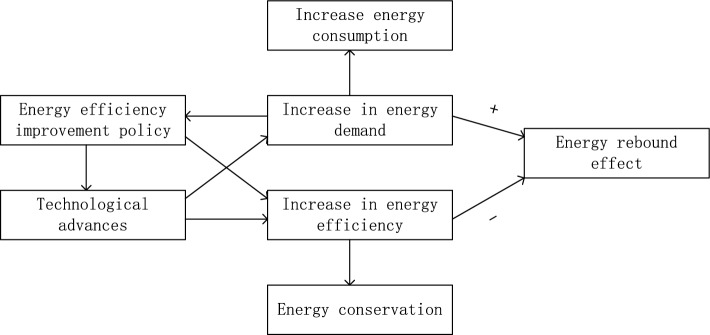
Structure diagram of the energy rebound effect.

Since 2013, China has launched carbon emissions trading pilots in seven provinces and cities, namely Beijing, Shanghai, Tianjin, Chongqing, Hubei, Guangdong, and Shenzhen. In December 2017, the National Development and Reform Commission officially announced the construction of a national carbon emissions trading system. On 16 July 2021, the nationwide carbon market was officially traded, with the power sector being the first to be included, making China the world's largest carbon trading market. As a market-oriented environmental regulation, the CETS makes use of a flexible market mechanism to encourage the leading carbon trading market players to innovate in a green way and keep their market edge^5^. Meanwhile, high-carbon enterprises are compelled to reduce carbon emissions, thus easing the pressure on production^6^. With the implementation of the CETS, a few scholars have already studied the effect of the CETS on EE and concluded that the CETS can significantly improve EE in China^7,8^. However, the improvement of EE may generate new energy demand through the mechanisms of the substitution effect, income effect and output effect, making the saved energy partially, completely, or even excessively consumed, thus generating an energy rebound effect^9^. The specific mechanism is shown in Fig. 1. The existence of the energy rebound effect implies that the implementation effect of the policy has not been brought into full play, and the rebound energy consumption will also significantly discount the carbon emissions that should have been reduced, which will ultimately affect the carbon reduction effect of the CETS. Some scholars believe that China's current ideas of energy efficiency-oriented policies are effective to a certain extent, but a considerable part of the potential effect of energy-saving and emissions reduction has not been realized, and there is still a large potential for energy-saving and emission redution^10^. The ultimate goal of the CETS to improve EE is also to reduce the use of fossil energy and to achieve carbon and energy separation. Therefore, this paper focuses on the fundamental purpose of the implementation of the CETS, and starts from the perspective of the rebound effect of energy-related carbon emissions (RECE) to reveal the actual carbon reduction effect of the CETS through the improvement of energy efficiency. The so-called energy-related carbon emissions rebound refers to the carbon emissions generated by the part of the energy that rebounds. The practice of this study is, on the one hand, a new perspective to examine the actual carbon reduction effect of the CETS, and on the other hand, it has significant practical implications for improving CETS.

This study will try to answer the following questions: Can the CETS improve EE? If the CETS can improve EE, what are the underlying mechanisms? During the implementation of CETS, is there RECE in the pilot provinces and cities, and what is the ultimate effect of carbon reduction through the improvement of energy efficiency? This paper uses the data of 30 provinces and cities (excluding Tibet Autonomous Region) in mainland China from 2007 to 2020 as the research sample to investigate whether the CETS can improve EE by using the difference-in-differences (DID) method, and examines the improvement path of EE from three aspects: government governance level, green innovation and industrial structure optimization. Finally, by calculating the national RECE, we compare and analyze the evolution of the RECE in six policy implementation pilots, and use it as an aid to judge the effectiveness of the policy implementation of the CETS.

The marginal contribution of this study is reflected in the following: first, single-factor energy efficiency (SFE) and total-factor energy efficiency (TFE) are used to test the research on the effect of CETS on EE, for which both measured by using the SBM-Malmquist-Luenberger index, avoiding the singularity of the factors in the traditional way of EE measurement. Secondly, unlike previous studies^11–13^, this study for the first time includes the RECE, skillfully combines energy and carbon emissions, and explores the impacts and consequences of the existence of the rebound effect on the actual carbon-reduction effect of the CETS that improves energy efficiency, further answering the question of the actual carbon reduction effect of the CETS through the improvement of energy efficiency. It enriches existing research on carbon trading system. Thirdly, the impact mechanism of the CETS to enhance EE is analyzed from the level of government governance, green innovation, and industrial structure optimization, which enriches the research on the path of the CETS to exert institutional dividends. In addition, it innovatively incorporates the level of government governance for the first time, to explore the intrinsic mechanism of the CETS to enhance EE, so as to provide references and bases for the government to guarantee the good operation of the carbon trading market.

The remaining portions of the essay are organized as follows: “Literature review and research hypotheses” is the literature review and research hypotheses. “Study design” is the research design. “Empirical results and analysis of impact mechanisms” is the empirical results and impact mechanism analysis. “Further research” is further research. “Research conclusions and policy recommendations” is the research conclusion and policy recommendations.

## Literature review and research hypotheses

### Literature review

#### Study of environmental regulation on energy efficiency

The green paradox argues that environmental regulation fails to achieve the desired energy savings^14^ through its regulatory effects^15^, but instead stimulates the quickening of fossil energy extraction and the overproduction of products that consume more energy. The widespread consensus is that environmental regulation inevitably raises production costs for firms, generating a cost compliance effect^16^ and crowding out funds available for R&D and innovation, to the detriment of advanced technological upgrading and energy efficiency^17^. Likewise, the incomplete enforcement of environmental regulations is an important reason for the green paradox as well, since the strength of environmental regulations is a trade-off and choice between economic development and environmental quality for local officials^18^, especially in the context of economic growth as a way to assess the performance of local officials, which will not only cause distortions in the structure of fiscal spending but also race to the bottom in environmental regulation^19^. Even worse, local officials are also inclined to introduce high energy-consuming industries, weakening the environmental regulatory intensity and lowering the barriers to foreign investment, leading to the pollution paradise effect^20^. Environmental regulations are relatively weak in developing countries and least-developed countries, becoming a refuge for carbon-intensive industries^21,22^.

Unlike the green paradox, the Porter hypothesis takes a different stance, which argues that environmental regulation might spur the regulated firms to make technological improvements such as production technologies modification, pollution control technologies innovation, and equipment upgrading to reduce the expense of environmental compliance, thus producing the innovation offset effect^23^. The cost compliance effect will be offset or even surpassed by this effect, thus generating gains in both production and energy efficiency, creating a unique competitive advantage relative to unregulated firms^24,25^. A reasonable environmental regulation not only stimulates firms to innovate green technologies^26^ and promote changes in their product mix^27^ but also pushes the industrial structure to upgrade^28^ and reduce the demand for fossil energy. In addition, environmental regulation can also promote EE by directing corporate investments and expanding technological and financial investments^29^.

However, studies by several academics have demonstrated that the correlation between environmental regulation and EE is complex and nonlinear rather than monotonous and linear. Among studies in this area, most of them show a U-shaped relationship between the two^30^, and environmental regulations above a certain threshold can compel enterprises to innovate new technologies and boost EE. The effect of different environmental regulation thresholds on EE varies, while factors such as foreign direct investment^31^, financing constraints^32^, and environmental decentralization^33^ are also important factors that influence this nonlinear relationship.

#### Research on the policy effects of CETS

The carbon emissions reduction impact has gained scientific attention since the CETS were put into place. As a proxy variable to study the carbon emissions reduction effect of the CETS, the attention to carbon emissions intensity has been rising. Most scholars' researches have proved that CETS can reduce carbon dioxide intensity to a great extent in pilot cities and exert a carbon emissions reduction effect. This policy effect can be positively influenced by elements like technological innovation, industrial restructuring, investments from abroad, and the degree of environmental regulation^34^. Second, since almost all greenhouse gas emissions come from the energy sector, it is clear that improving EE is essential for reducing carbon emissions^35^. Some studies have shown that improving EE is necessary to achieve the decoupling of economic growth from carbon^36^. Some scholars' studies prove that carbon emissions trading can improve EE through the channels of adjusting energy structure, promoting green technological innovation, and rational allocation of resources^7,8^. Meanwhile, as a market-oriented environmental regulation tool, its corresponding economic effects and social welfare have also received attention and examination. A few studies have demonstrated that the CETS can improve the investment efficiency of enterprises^37^ and the allocation efficiency of capital^38^, and enhance the economic benefits of township enterprises in pilot cities^39^. It has also been found that the CETS will show a negative impact on the overall economy, but it will be gradually eliminated in the long term^40^, and the CETS can partially compensate for the loss of GDP caused by carbon emission reduction^41^. Furthermore, the CETS can change the employment structure, enhance the employment share of the highly educated labor force^6^, and broaden the employment scale ^42^.

#### Research on the energy rebound effect

There is a complex correlation between EE and the energy rebound effect, where the lack of EE improvement is a cause for the energy rebound effect^43^. On the other hand, energy consumption will rise rather than fall as a result of EE advancements, and increases in demand for services without increasing fuel prices will deplete the gains in technical efficiency^44^. The demand for energy services will rise resulting from substitution, income, and output effects brought on by a drop in the effective price of energy services^45^.

The energy rebound effect has been measured in existing studies in terms of the overall economy, industry, and sector^46,47^, but the measurement results vary widely due to different assumptions of premises, model settings, and estimation methods. In addition to being directly influenced by energy efficiency, the effect is also indirectly influenced by the degree of industrial linkage^48^, marketization^49^, urbanization, investment preferences, factor market distortions, and administrative decentralization^20^. Some studies also find that digital development, industrial robotics, and information and communication technology trigger energy rebound effects that offset some of the carbon reduction effectiveness^50,51^. Wang and Wang consider improving EE and expanding trade openness as two important measures to counteract the vengeful rebound of carbon emissions post-COVID-19 pandemic^35^. In addition, market-oriented and complementary policies such as prices and taxes need to be introduced to diminish the energy rebound effect^52^. Some scholars have considered the rebound effect in the process of exploring the implementation effect of CETS. Chen et al. further estimated the energy rebound effect on the basis of confirming that the CETS can improve China's EE, and concluded that the energy rebound effect of the pilot provinces was higher than that of the non-pilot provinces during 2000–2017^12^. Li et al. found that the implementation of the CETS had a partial rebound effect on the decarbonization of the power sector in China^53^. Further, Bolat et al. confirmed the macroeconomic carbon rebound effect of the EU Emission Trading System (ETS), suggesting that positive economic spillovers from the ETS may hinder the achievement of climate goals^54^.

The above findings indicate that the relationship between environmental regulation and energy efficiency is still controversial. As a market-based environmental regulation, the relationship between the CETS and EE needs to be further verified, and the corresponding studies have not explored its influence mechanism from the perspective of the level of government governance. In addition, most of the current studies on the impact of CETS on EE have not yet considered the rebound effect, and the conclusions obtained are simple promotional effects. However, discussing the role of CETS in promoting EE independently of the rebound effect may deviate from the actual situation. Although individual scholars have considered the energy rebound effect in their studies on the impact of CETS on EE, they have not compared and analyzed the average energy rebound effect in the two time periods before and after the implementation of the policy, and the conclusions do not include the specific practical effects after the implementation of the policy. Therefore, based on the above research results, this study for the first time incorporates the level of government governance to explore the intrinsic mechanism of CETS affecting EE. Secondly, the study starts from the perspective of the RECE for the first time, which is a unique combination of energy consumption and carbon emissions, and explores the corresponding consequences of its existence on the improvement of EE. Returning to the essence of the implementation of the CETS, the research question forms a complete closed loop.

### Research hypotheses

As an environmental tool with the advantages of low economic cost, high flexibility, and continuous improvement^55^, the CETS is a favorable strategy to separate the expansion of the economy from the increase in carbon emissions. With emission rights as the carrier, the local officials issue free allowances according to the emission reduction target, and enterprises with advanced technology and lower emission reduction costs can sell out the extra carbon quota to enterprises with backward technology for reducing emissions, thus gaining economic benefits and encouraging and forcing enterprises to improve EE to realize carbon emissions reduction. In this process, the higher the level of government governance, the more motivated enterprises are to join in carbon emissions trading^56^. This is because a highly-governed government can ensure the existence of a regulated and well-run carbon market, stabilize the carbon price and guarantee the compliance of emission-controlling enterprises' quotas by strengthening quota management, speeding up administrative approval, and strengthening market supervision. Moreover, improving relevant laws and regulations and establishing a transparent and perfect carbon trading market system, greatly reduces the transaction costs of enterprises and stimulates their passion to take part in carbon trading^57^. Thus, it will stimulate enterprises to continuously strengthen technological innovation to reduce carbon emissions, optimize production processes, improve production technologies and eliminate outdated productivity, and ultimately improve EE. Given this, this paper proposes:

H1: The optimization of the government governance level can help to enhance the promotion of EE by the CETS.

Green innovation is a powerful tool for increasing EE and achieving low-carbon economic development since it can boost technological efficacy and cut greenhouse gas emissions. Reasonable environmental regulations can push companies to green innovation and reduce their environmental compliance costs^58^. A carbon emissions trading system uses a price mechanism to reward low-carbon emitting firms for selling their excess carbon allowances to gain excess revenue and penalize high-carbon emitting firms for bearing excess carbon emissions^59^. Relying on the flexible market mechanism to internalize environmental costs, the dominant enterprises are encouraged to continuously develop green and low-carbon technologies to stabilize their competitive advantages, while the disadvantaged enterprises are compelled to perform research and development of clean technologies to compensate for their carbon emission reduction costs with the innovation compensation effect. Second, the government alleviates the financial pressure on enterprises through direct subsidies, tax incentives, and green credits to support their green innovation^7,60^, improve energy efficiency, and help them achieve green and low-carbon transformation. Based on this, this paper proposes that:

H2: CETS can improve EE by promoting green innovation.

At present, China's industrial structure still struggles with the issue of unbalanced development, and the secondary industry remains a key pillar of China's economic development, but it is also the main driver of energy consumption and carbon emissions, and its energy efficiency is relatively backward. Optimization of industrial structures is a crucial tactic for increasing EE^61^, and reasonable environmental regulation can guide the industrial structure to change to low carbon and reduce energy use intensity. Firstly, by altering the relative prices of resource factors^62^, setting barriers to market access, and supporting the growth of environmentally preferred industries^63^, environmental regulation can have an impact on the allocation of resource factors across different sectors, the elimination of unnecessary industries, and the encouragement of industrial structure optimization. Secondly, unlike a carbon tax, the CETS has specific carbon reduction targets. Subjected to this emission reduction target, the original energy-consuming industries, to achieve sustainable development and get rid of high energy dependence, often choose to transform to industries with low carbon emissions, such as the service industry and high-tech industry, making industrial structure change and economic development mode transform^64^. Based on this, this paper proposes:

H3: CETS promotes EE by guiding industrial structure optimization.

## Study design

### Variable definition and data sources

#### Explained variables

This paper chooses energy efficiency(EE) as an explained variable, which is separated into single-factor energy efficiency (SFE) and total-factor energy efficiency (TFE), and is measured by using the SBM-Malmquist-Luenberger index concerning Emrouznejad and Yang^65^ and Huang,et al.^66^. In this case, the TFE uses energy, labor, and capital as inputs, gross regional product (GDP) as consensual output, and carbon emissions as non-consensual output. The SFE is measured in the same way, but the factor effects of labor and capital are excluded, and only the energy factor is measured as an input. (1) energy is characterized by total provincial energy consumption; (2) labor is expressed by the number of employees per unit at the end of the year; (3) capital is measured by capital stock, which is estimated by the perpetual inventory method based on the formula ${K}_{t}={K}_{t-1}\left(1-\delta \right)+{I}_{t}$, where ${K}_{t}$ is the base stock in the base period, ${K}_{t-1}$ is the capital stock of the previous period, δ is the depreciation of fixed assets, the capital depreciation rate is set at 10.96%, and ${I}_{t}$ is the total investment in fixed assets. (4) Carbon emissions: according to the United Nations Intergovernmental Panel on Climate Change^67^, the energy consumption data of each province by category, specifically coal, coke, gasoline, crude oil, diesel, kerosene, fuel oil, liquefied petroleum gas, natural gas, and electricity, are converted according to carbon emission factors and aggregated to obtain provincial carbon emissions. To eliminate the effect of inflation, the monetary variables involved in the above variables are deflated by the base period.

#### Channel variables

1. Government governance level (GGL).
  Referring to the study of Wang et al.^68^, the government governance level indicator system is constructed from three aspects: business environment, private economic development level and innovation environment (Supplementary Table S1), and the entropy value method is used to calculate the evaluation results.
2. Industrial structure optimization (ISO).
  In this paper, industrial structure advance is chosen as a proxy variable for industrial structure optimization and uses the ratio of the output value of the tertiary industry to that of the secondary industry to measure it. The larger the ratio, the more the industrial structure shifts from secondary industry dominance to tertiary industry, and the way economic development moves towards high-quality development.
3. Green innovation (GI).
  Green innovation (GI) is measured by the ratio of the number of green invention patents granted to the resident population of the region (pieces/1000 people)^69^.

#### Control variables

The control variables include population density, which is measured by dividing the provincial population by the administrative area. Structural transformation is characterized by the proportion of the output value of the tertiary industry and the output value of the primary and secondary industries. Energy consumption is obtained by converting the energy consumption of each province into ten thousand tons of standard coal. The industrial structure is measured by dividing the added value of the secondary industry by the GDP. The urbanization level is obtained by dividing the urban population by the resident population at the end of the year.

The above data were obtained from the National Bureau of Statistics, China Energy Statistical Yearbook, China Population and Employment Statistical Yearbook, China Science and Technology Statistical Yearbook, wind financial database, and CSMAR database.

The descriptive statistics of the main variables are shown in Table 1, where the mean value of the dependent variable SFE is − 1.114, the minimum is − 4.071, the maximum is 0.466, and the standard deviation is 0.656. The mean value of TFE is − 0.799, the minimum is − 1.949, the maximum is 0.466, and the standard deviation is 0.421, suggesting that both the statistical results of the remaining control variables are consistent with existing studies.

**Table 1:** Descriptive statistics of main variables.

Variable symbol	Variable name	Number of observations	Mean	Standard deviation	Minimum	Maximum
SFE	Single-factor energy efficiency	420	− 1.114	0.656	− 4.071	0.466
TFE	Total-factor energy efficiency	420	− 0.799	0.421	− 1.949	0.466
Density	Population density	420	7.860	0.436	6.433	8.694
Structure	Structural transformation	420	0.919	0.615	0.401	5.199
Energy	Energy consumption	420	9.351	0.686	6.963	10.641
Industry	Industrial structure	420	0.404	0.089	0.159	0.601
Urban	Urbanization level	420	0.563	0.134	0.282	0.896

### Model construction

#### Difference-in-differences model design

In this paper, 2007–2020 is chosen as the sample period, due to the data availability, 30 provinces in mainland China (except Tibet) are selected as the research sample. China's carbon trading pilot policy has been implemented since 2013, so we have taken 2013 as the base year for the implementation of the policy. 2013–2020 is set as the executing year of the CETS, and 2007–2012 is taken as the period prior to the implementation of this policy. For the division of experimental and control groups, Shenzhen is included in Guangdong Province, and six provinces and cities with CETS are used as the experimental group, of which the remaining provinces are used as the control group. To assess the effect of CETS on EE, this study utilizes the DID method for policy evaluation, and then constructs the following model:

1 $${EE}_{it}={\alpha }_{0}+{\alpha }_{1}\left({treat}_{it}\times {period}_{it}\right)+\delta {Controls}_{it}+{\gamma }_{i}+{\theta }_{t}+{\epsilon }_{it}$$

where i and t represent province and year, respectively. EE stands for SFE and TFE, respectively. Treat denotes the province grouping variable, 1 for pilot provinces of CETS and 0 for non-pilot provinces. Period is the time grouping variable, 1 for 2013–2020, and for 2007–2012 is 0. Controls are the set of control variables. γ is the province-fixed effect that does not vary with time. θ is the time-fixed effect. ${\epsilon }_{it}$ is the random error term. The impact of the CETS on EE is estimated mainly by observing the coefficients of treat × period.

#### Model construction of impact mechanism

1. Mediating effect model.
  This study chooses the mediating effect model to test the mediating role played by GGL and GI, and takes a reference to Baron and Kenny^70^ to test the significance of coefficients in three steps in turn, to examine whether the mediating effect holds. The remaining two test models are constructed after the baseline regression:
  2 $${Mediator}_{it}={\beta }_{0}+{\beta }_{1}\left({treat}_{it}\times {period}_{it}\right)+\rho {Controls}_{it}+{\gamma }_{i}+{\theta }_{t}+{\epsilon }_{it}$$
  3 $${EE}_{it}={\varphi }_{0}+{\varphi }_{1}\left({treat}_{it}\times {period}_{it}\right)+{\varphi }_{2}{Mediator}_{it}+\vartheta {Controls}_{it}+{\gamma }_{i}+{\theta }_{t}+{\epsilon }_{it}$$
  In the above equation, Mediator represents the mediating variable, specifically referring to GGL and GI, and the significance of treat × period and Mediator coefficients are mainly examined to determine whether they are fully or partially mediated effects^71^, and the remaining variables are following Eq. (1).
2. Moderating effect model.
  This study mainly takes a reference to the study of Wang et al.^72^ and embeds the ISO variables affecting EE into Eq. (1) to examine the significance level of the influence mechanism.
  4 $${EE}_{it}={\omega }_{0}+\omega \left({treat}_{it}\times {period}_{it}\times {Moderator}_{it}\right)+{\omega }_{2}\left({treat}_{it}\times {period}_{it}\right)+ {\omega }_{3}{Moderator}_{it}+\tau {Controls}_{it}+{\gamma }_{i}+{\theta }_{t}+{\epsilon }_{it}$$
  In this equation, Moderator represents the moderating variable ISO, and the significance of the treat × period × Moderator coefficient is mainly examined. The original industry structure control variable is excluded from the regression with ISO as the moderating variable to avoid the covariance problem, and the remaining variables are defined in accordance with Eq. (1).

#### Energy carbon emission rebound effect model based on technological progress

Hu et al.^73^ used two different rebound effect measures to estimate the energy rebound effect in Chinese cities, one is the rebound effect based on energy-enhanced technological progress proposed by Saunders^74^ and the other is the rebound effect based on generalized technological progress proposed by Berkhout^75^, and the comparative analysis found that the rebound effect of Berkhout is more applicable to the measurement and study of the rebound effect of a single subject variable and a small number of multiple subjects. Since this study selects the data at the provincial level as the sample, there are relatively few subjects to measure, so the method proposed by Berkhout is chosen to measure the RECE in China.

Assuming that the regional GDP of an economic unit in year t is ${Y}_{t}$ and the energy-related carbon emissions intensity is ${{\text{CI}}}_{{\text{t}}}$, the energy-related carbon emissions in year t is ${C}_{t}={Y}_{t}\cdot {CI}_{t}$. In year t + 1, due to technological progress, the energy-related carbon emissions intensity decreases to ${CI}_{t+1}$. Then, the reduction in energy-related carbon emissions due to technological progress in year t + 1, ${C}_{j}$ is calculated as:

5 $${C}_{j}={Y}_{t+1}\cdot ({CI}_{t}-{CI}_{t+1})$$

Technological progress must lead to economic growth, and if the contribution of technological progress to economic growth in year t + 1 is ${\sigma }_{t+1}$, then the rebound of energy-related carbon emissions (${C}_{k}$) in year t + 1 is:

6 $${C}_{k}={\sigma }_{t+1}\cdot \left({Y}_{t+1}-{Y}_{t}\right)\cdot {CI}_{t+1}$$

where ${\sigma }_{t+1}$ is calculated as:

7 $${\sigma }_{t+1}=\frac{{G}_{TFP}}{{G}_{Y}}=\frac{\frac{{TFP}_{t+1}-{TFP}_{t}}{{TFP}_{t}}}{\frac{{Y}_{t+1}-{Y}_{t}}{{Y}_{t}}}$$

Therefore, the rebound effect of energy-related carbon emissions in year t + 1 is:

8 $$RE=\frac{{C}_{k}}{{C}_{j}}={\sigma }_{t+1}\cdot \frac{({Y}_{t+1}-{Y}_{t}){CI}_{t+1}}{{Y}_{t+1}({CI}_{t}-{CI}_{t+1})}$$

When RE < 0, it refers to a super-saving effect, which indicates that the significant enhancement of EE reduces energy consumption and carbon emissions, and the relevant EE improvement policies are effective at this time.

When RE = 0, it refers to zero rebound effect, which means that energy consumption does not increase with EE, and the corresponding carbon emission does not increase with it, which is equal to the expected value, reflecting the effectiveness of EE improvement strategies.

When 0 < RE < 1, it refers to partial rebound effect, which means that as the energy consumption increases with the improvement of EE, the corresponding carbon emission increases, but the increased carbon emission is lower than the reduction amount, so it is a partial rebound effect.

When RE = 1, it refers to full rebound effect, which means that the improvement of EE increases energy consumption, thus generating more carbon emissions which is equal to the reduction amount, and the EE improvement policy is not effective at this time.

When RE > 1, it refers to tempering effect, which means that with the improvement of EE, the carbon emission generated exceeds the reduction amount, and the EE improvement policy shows a negative effect.

## Empirical results and analysis of impact mechanisms

### Parallel trend test

**Figure 2:**
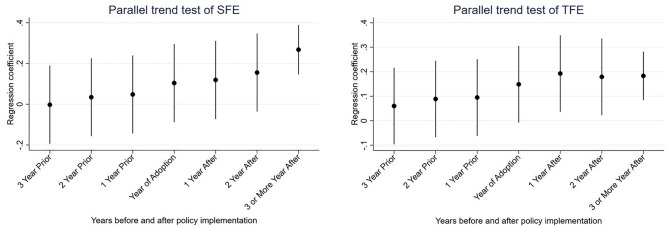
Parallel trend test results.

The parallel trend assumption, which states that both SFE and TFE retain a comparatively stable trend of change prior to the pilot installation of the CETS, is satisfied by the control and experimental groups when using the DID method. In this paper, the OLS-DID regressions were conducted for SFE and TFE in line with the main regression, using 2013 as the base year, and the sample data of three years before and three years after this base year and above were selected. The results, listed in Fig. 2, find that the regression coefficients of treat × period are not significant in the first three years of the CETS pilot, which is consistent with the assumption of a parallel trend.

### Baseline regression results

Table 2 reports the regression results. The regression results in group (1) reveal that the coefficients of treat × period are positive and significant at the 1% level after controlling for the time and province effect, and the preliminary judgment is that the CETS can significantly improve EE of the pilot provinces. When adding control variables in group (2), the coefficients of treat × period drop but the significant level remains unchanged, which indicates that the CETS can significantly improve the SFE and TFE of the pilot provinces. Meanwhile, the model's goodness of fit grows with the addition of control variables, indicating that the model fit is subsequently improved.

**Table 2:** Carbon emissions trading system and energy efficiency: DID regression results.

Variables	(1)		(2)	
	SFE	TFE	SFE	TFE
Treat × period	0.201*** (4.91)	0.139*** (4.70)	0.110*** (3.03)	0.090*** (2.70)
Industry			0.231 (0.95)	0.035 (0.20)
Energy			− 1.143*** (− 17.22)	− 0.612*** (− 28.58)
Density			− 0.024 (− 0.92)	0.092*** (2.93)
Urban			2.149*** (3.57)	0.774** (1.99)
Structure			0.205 (1.15)	0.008 (0.19)
cons_	− 1.137*** (− 113.52)	− 0.815*** (− 103.16)	8.262*** (9.70)	3.730*** (7.76)
Year	Yes	Yes	Yes	Yes
Province	Yes	Yes	Yes	Yes
N	420	420	420	420
Adj-R^2^	0.921	0.873	0.978	0.911

Values in parentheses are t-values, and ***, **, and * indicate 1%, 5%, and 10% significance levels, respectively, as follows.

Among the control variables, energy consumption suppresses SFE and TFE at the 1% level. Population density increases TFE at the 1% level, which may be due to the fact that population clustering increases the share of highly qualified personnel and moves to energy-efficient industries, fully utilizing the demographic dividend. The level of urbanization significantly increases SFE and TFE, and the coefficients of other control variables are not significant, indicating that they are not the primary factors affecting EE.

### Robustness tests

#### Placebo test

**Figure 3:**
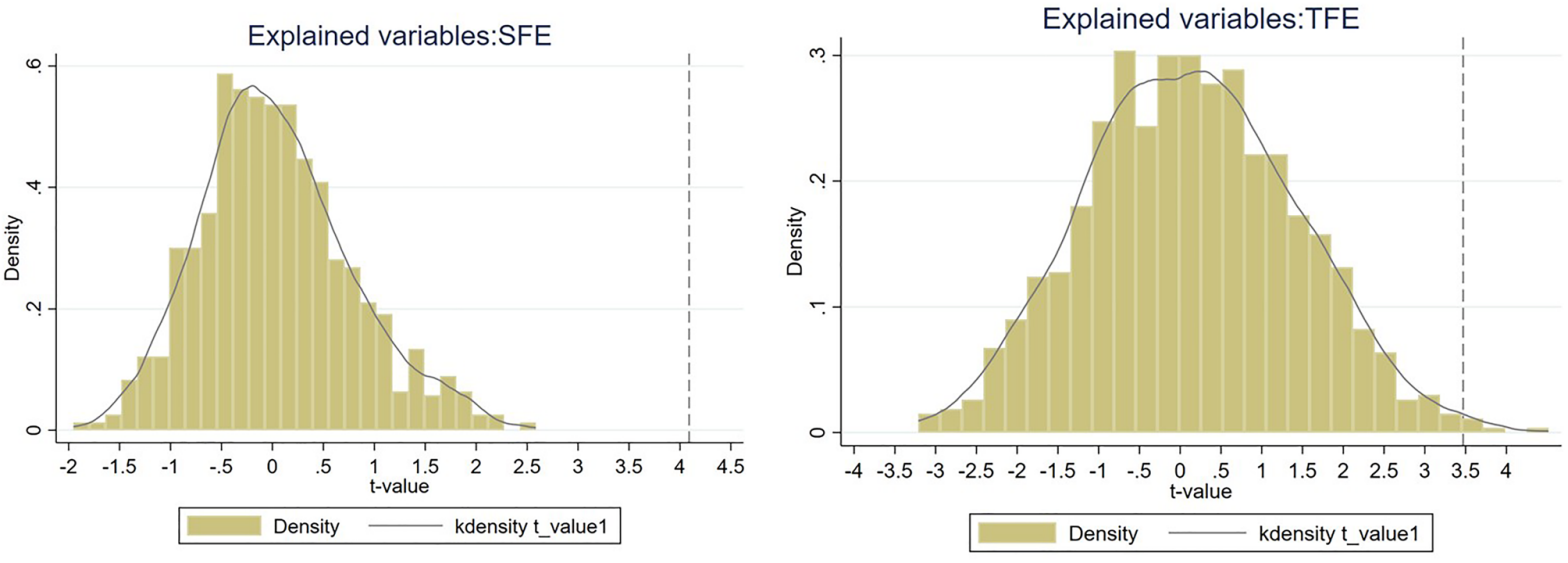
Placebo test results.

The effect of the CETS on EE may be disturbed by other unknown factors. To exclude the influence of these uncertainties and ensure that EE is caused by the CETS, a placebo test is required. In this paper, six provinces out of 30 provinces are chosen at random to represent the experimental group, and the remaining are used as the control group, with SFE and TFE as the dependent variables, and 1000 random sampling and DID regressions are conducted respectively. Figure 3 shows the kernel density estimation plots of the t-values of the two energy efficiencies. The results show that the means of the kernel density estimates of both the randomized coefficients and the t-values deviate significantly from their true values, proving that the results hold up to a placebo test. This means other unobservable factors or missing variables are not responsible for the CETS' impact on EE.

#### Robustness test based on the year of replacement policy implementation

Considering that the CETS was implemented in each pilot city successively from 2013 to 2014, there is a certain lag of policy in effect. To improve the robustness of the findings, this study takes 2015 as the base year of policy implementation and conducts regressions according to the DID model. Supplementary Table S2 shows that the results are consistent with those of using 2013 as the base year, adding to the evidence that the study's conclusions are solid.

#### Dynamic time window test

This paper draws on Shi and Li^76^ to test the magnitude of the difference in the impact of the CETS on EE over different periods by varying the width of the time window before and after the policy introduction. This is done by taking 2013 as the time point of policy introduction and selecting 1, 2, 3, and 4 years as the time window widths. According to Supplementary Table S2, as the time window width changes, the CETS' impact on EE is not affected in a particular way, and the coefficients and significance keep increasing, indicating the robustness of the conclusions.

#### PSM-DID test

Since the DID method has a selectivity bias and cannot achieve the ideal quasi-natural test effect, the regression findings are checked and verified for accuracy using the score-dumping matching method. Specifically, a logit model was used to select industrial structure, population density, urbanization level, and structural transformation as matching variables, and the experimental group was matched with the control group using caliper matching to reduce the selective bias caused by individual differences. According to the matching effect test criterion proposed by Rosenbaum and Rubin^77^, if the absolute value of the standard deviation of the sample variables after matching does not exceed 20%, the matching effect is good and the matched sample estimates are true and valid. The findings in Supplementary Table S3 are consistent with this criterion, and all of the p-values are higher than 10%, indicating that the matching results of the samples are reasonably valid.

The matched samples were subjected to DID estimation, and Supplementary Table S4 displays the results of the estimation. The CETS still significantly improves SFE and TFE, which indicates the validity of the conclusions obtained in this paper.

#### Controlling the impact of other energy efficiency improvement policies

In March 2016, China proposed for the first time to establish a green certificate trading mechanism (GCTM) for renewable energy power. It was officially launched in July 2017, becoming one of the alternative ways to subsidize renewable energy power generation. The GCTM is a crucial tool for enhancing the dual control of energy intensity and total amount, which is important to improve EE, promote clean and low-carbon energy transformation, and guarantee reasonable energy demand ^78^. To control the impact of GCTM on EE and accurately identify the impact of the CETS on EE, we collected purchase data from 30 provinces on the China Green Power Certificate Subscription Trading Platform and processed the data by taking the logarithm as a proxy variable for the green certificate trading system. A baseline regression consistent with the main regression was conducted by including the GCTM as a control variable, and Supplementary Table S5 provides the results. The coefficients of treat × period of the two main explained variables decrease, but the direction of influence remains unchanged and significant, indicating that the findings remain unchanged after eliminating other energy policy interferences.

### Analysis of the influence mechanism

The mechanism of the impact of the CETS on EE is examined using the model constructed above, with GI and GGL serving as mediating variables and ISO serving as moderating variables. The test findings are displayed in Table 3. Columns (1)–(3) and (4)–(6) of Table 3 test the mediating effects of GI and GGL between CETS and EE, respectively, and columns (7)-(8) test the moderating effect played by ISO.

**Table 3:** Results of impact mechanism test.

Variables	Mediating effect						Moderating effect	
	GGL	SFE	TFE	GI	SFE	TFE	SFE	TFE
	(1)	(2)	(3)	(4)	(5)	(6)	(7)	(8)
Treat × period × ISO							0.0866** (1.97)	0.0314* (1.83)
Treat × period	0.0467*** (6.12)	0.0622* (1.69)	0.0669** (2.04)	0.0080* (1.97)	0.1647*** (4.22)	0.1238*** (3.56)	− 0.0175 (− 0.23)	0.0383 (0.72)
ISO							− 0.0735 (− 1.08)	− 0.1379*** (− 2.84)
GI					3.5374*** (3.45)	0.8864* (1.85)		
GGL		1.0175*** (4.33)	0.4994*** (2.91)					
Cons_	− 0.0280** (− 0.12)	8.2908*** (11.66)	3.7446*** (7.58)	− 0.0723 (− 0.96)	− 1.3429* (− 1.67)	− 1.5353*** (− 2.64)	9.0796*** (13.06)	4.3395*** (9.25)
Controls	Yes	Yes	Yes	Yes	Yes	Yes	Yes	Yes
Year	Yes	Yes	Yes	Yes	Yes	Yes	Yes	Yes
Province	Yes	Yes	Yes	Yes	Yes	Yes	Yes	Yes
N	420	420	420	420	420	420	420	420
Adj-R2	0.9610	0.9803	0.9125	0.8969	0.9284	0.8732	0.9779	0.9121

1. Level of government governance.
  The results of columns (1)–(3) reflect that the CETS significantly improves GGL, and further improves EE through the improvement of GGL, which verifies hypothesis 1. In the pilot areas, in order to maximize the effect of the CETS, local governments will improve relevant laws and regulations, improve the level of supervision and management, rationally allocate regional resources and stabilize carbon prices^79^, to provide a good market environment for enterprises. The higher the GGL, the CETS can further improve EE.
2. Green innovation.
  Column (4) shows that the CETS promotes GI at a significant level of 10%, which is consistent with the findings of Xin et al.^80^. Columns (5)–(6) show that the coefficients of treat × period and GI are positive and significantly improve SFE and TFE, indicating that the CETS can improve EE through GI, and hypothesis 2 is confirmed.
3. Industrial structure optimization.
  Columns (7)–(8) show that ISO can significantly enhance the promotion effect of CETS on SFE and TFE. It verifies hypothesis 3 that ISO has a beneficial moderating effect between CETS and EE, which keeps the same as the study of Li et al.^1^ that industrial structure optimisation can intensify the role of environmental regulations in improving energy efficiency.

## Further research

### Measurement results of the rebound effect of energy-related carbon emissions in China from 2007 to 2020

Supplementary Table S6 shows the calculation results of RECE in China from 2007 to 2020, in which the ratio of years showing the super-saving effect and partial rebound effect is 6:8 respectively, which indicates that the RECE in China from 2007 to 2020 shows the partial rebound phenomenon in most years.

Among the sudden extreme events, the global financial crisis broke out in 2008, by which China’s carbon emissions dropped dramatically in 2009, and the RECE showed a super-saving effect, but it started to rise gradually from 2010 onwards and showed a partial rebound effect, which is consistent with the findings of Wang and Wang ^35^. In 2019, the COVID-19 epidemic broke out, and after the epidemic was gradually controlled in 2020, China began to resume work and production. In response to the epidemic, medical supplies, necessities, and construction materials started to be produced in large quantities in the short term, resulting in more energy consumption and a partial RECE.

### Comparative analysis of the rebound effect of energy-related carbon emissions based on carbon trading pilot

**Figure 4:**
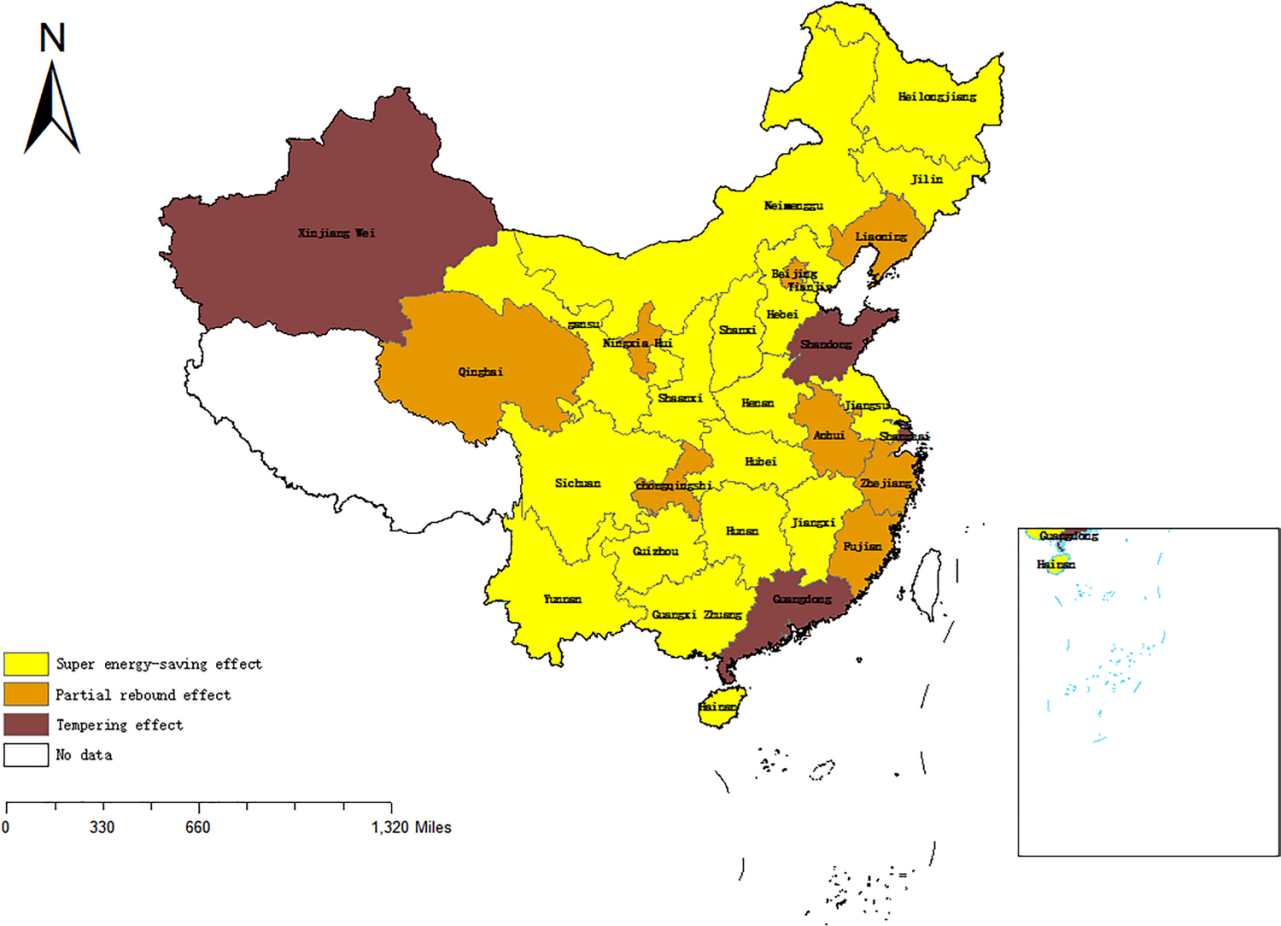
Rebound effect of energy-related carbon emissions in 30 Chinese provinces, 2007–2012. (No. GS(2020)4632).

**Figure 5:**
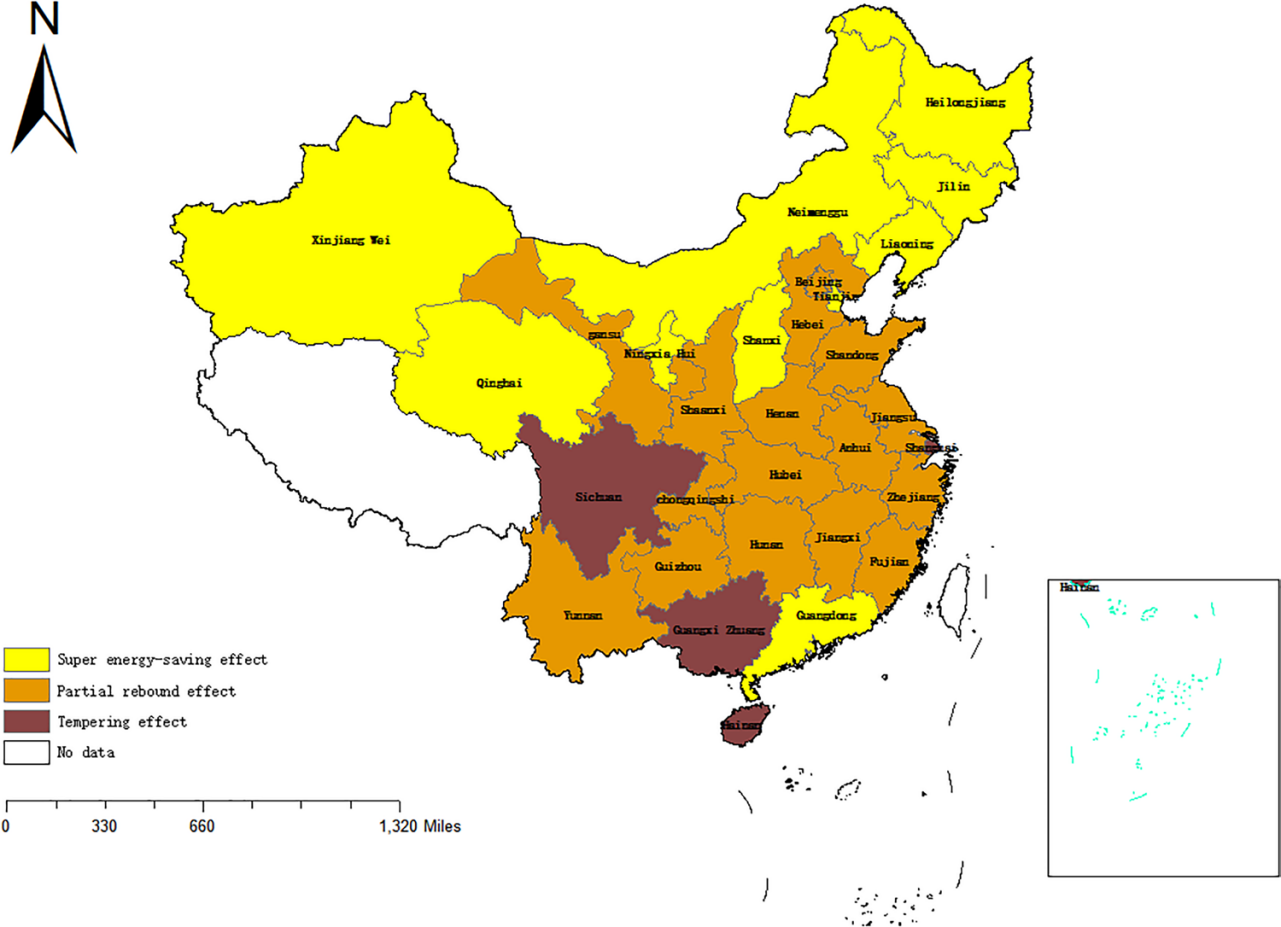
Rebound effect of energy-related carbon emissions in 30 Chinese provinces, 2013–2020. (No. GS(2020)4632).

In the pilot and non-pilot provinces and cities of China's CETS, Figs. 4 and 5 (drawn through ArcGIS 10.7: https://www.esri.com/zh-cn/arcgis/products/arcgis-desktop/overview) and Table 4 compare and analyze the differences in the RECE before and after the implementation of the policy. It is evident from Figs. 4 and 5 that the distribution ratio of RECE before and after 2012 is roughly the same, but the rebound effect of some provinces has changed, which makes a big difference in each pilot province. Among them, the rebound effect in Guangdong Province shifted from a tempering effect to a super-saving effect. This indicates that the actual energy consumption is less than its expected value, and the corresponding reduction in carbon emissions is greater than the expected value, which suggests that the CETS shows significant results. In Hubei, the rebound effect changes from a tempering effect to a partial rebound effect after the policy implementation, which indicates that the carbon emissions reduced by improving energy efficiency will partially rebound. In Tianjin, the rebound effect before and after the implementation of the policy is a super-saving effect, and the CETS will have a chain reaction by improving energy efficiency, which will have more carbon reduction effects than expected. However, Beijing, Shanghai and Chongqing all have partial rebound effects before and after the implementation of the policy, which will partially weaken the effectiveness of the policy. The local governments of these places should give due consideration to the impact of RECE when formulating policies to improve EE, and ought to stabilize or increase the effective price of energy to curb RECE.

**Table 4:** Comparison results of grouping the rebound effect of energy-related carbon emissions.

	Pilot provinces	Non-pilot provinces	All provinces
2007–2012	0.329	− 0.318	− 0.415
2013–2020	0.314	0.095	0.061
2007–2020	0.321	− 0.082	− 0.143

The mean value of the RECE in China during 2007–2020 was − 1.43%, showing a super-saving effect. The possible reason for this is the influence of extreme emergencies, such as the financial crisis in 2008 and the new crown epidemic in 2019, which led to a super-saving effect in the mean value of the RECE in China over the study period. In addition, referring to the study of Zhang et al.^81^, China's energy rebound effect was − 1.42% in 1982–2009, of which several years after 2003 showed a super-saving effect, corroborated with the super-saving effect showed in 2007–2009 in our study. After the policy’s implementation, the RECE of pilot provinces showed a 4.56% decrease during 2013–2020, but still higher than the non-pilot provinces, showing a partial rebound effect. This is due to the rebound of the expected reduction in carbon emissions as a result of the increased EE of the CETS, which stimulates more energy demand^12^. The rebound effect for the pilot provinces was 0.314, indicating that 31.4% of the carbon emissions reduced through EE improvements rebounded in 2013–2020. The rebound effect in the pilot provinces was 0.314, indicating that 31.4% of the carbon emissions reduced through EE improvements rebounded, implying that the CETS was only able to achieve 68.6% of the carbon reduction effect through EE improvements. The potential of the CETS to reduce carbon emissions by improving EE has not been fully realized, and the EE improvement policies still need to be further improved.

## Research conclusions and policy recommendations

The DID model is used in this study to illustrate how CETS affect EE by dividing EE into SFE and TFE. At the same time, the role path of the CETS to improve EE is explored from three perspectives: government governance level, green innovation and industrial structure optimization. Finally, by calculating the RECE in each province, the corresponding evolution of the overall and pilot provinces is compared and analyzed as an auxiliary tool to judge the effectiveness of CETS. The main findings are as follows: ① After a series of robustness tests such as parallel trend test, placebo test, replacement of policy implementation years, dynamic time window test, PSM-DID, and controlling the impact of other energy policies, it is shown that the CETS can greatly enhance SFE and TFE in the pilot provinces and cities. ② The verification of the influence mechanism indicates that the CETS can greatly enhance SFE and TFE through the improvement of government governance level, green innovation, and industrial structure optimization. ③ The macro-level RECE in China from 2007 to 2020 was − 1.43%. The RECE reduction in pilot provinces decreased by 4.56% compared to before the policy's implementation, but it remained considerably higher than that in non-pilot provinces. The RECE in the pilot provinces was 31.4% from 2013 to 2020, implying that the CETS can only achieve 68.6% of the carbon reduction effect by improving EE, and the CETS has yet to realize its full carbon reduction potential. The following specific policy recommendations are made in this report based on the aforementioned study findings:

1. Actively improve China's carbon emissions trading system, promote the construction of a unified carbon emissions trading market in China, and gradually include more industries, enterprises and market players to participate in carbon trading. Expand the market scale, improve the surplus adjustment capacity of allowances, and alleviate the trend of slowing down or even decreasing the rate of carbon emission allowances growth.
2. Actively pay attention to the corresponding changes in China’s RECE during the implementation of the CETS, and use it as an auxiliary tool to judge the effectiveness of the policy and dynamically adjust the carbon emissions reduction constraint target. It is also supplemented by other energy efficiency improvement policies and instruments, absorbing the advanced experience in the years of excessive storage effect, and reflecting on and improving the years of tempering effect to leverage policy synergies.
3. Strive to improve government governance and encourage green innovation of emission reduction subjects. By perfecting relevant laws and regulations, the rights and obligations of all participants are clarified. Formulate and improve relevant rules against unfair competition and anti-monopoly to enhance market liquidity and activity. At the same time, strengthen market supervision, increase incentives and penalties, enhance compliance guarantee, and improve the reliability of carbon emission trading reports. Optimize corporate financing channels, reduce corporate green innovation risks and financial constraints, and mobilize corporate green innovation initiatives in a backward and auxiliary manner.
4. The industrial structure should be adjusted and optimized with an emphasis on resource allocation and technological advancement. The proportion of clean energy and renewable energy sources ought to increase, and structural reform on the side of energy supply from the source is supposed to be encouraged. Furthermore, a reasonable industrial policy is formulated to play a synergistic part in the CETS to guide the low-carbon transformation of energy-consuming industries and improve EE.

### Supplementary Information

Supplementary Tables.

## Data Availability

Data will be made available from the corresponding author on reasonable request.
