# Supplementary material for: Practical effects of carbon emissions trading system on energy efficiency
Source: Sci Rep. 2024 Jan 2;14:279. doi: 10.1038/s41598-023-50621-3 (PMC10761875; doi:10.1038/s41598-023-50621-3)
Supplement: Supplementary file 1 — Supplementary Tables. [file 41598_2023_50621_MOESM1_ESM.docx]

Supplementary Material

**Does the carbon emissions trading system promote energy efficiency？ Evolutionary Perspective Based on the Rebound Effect of Energy Carbon Emissions**

*Xue Li^1^, Aochen Cao^2^, Yuhan Zhang^1^, Yuting Zhao^1^, Lulu Chen^1^, Pan Jiang^1^, Liang Liu^1^*

^1^ School of Economics and Management, Southwest University of Science and Technology, Mianyang, 621010, China

^2^ School of Economics, Central University of Finance and Economics, Beijing, 102206, China

Corresponding Author

*Author to whom correspondence should be addressed:

Liang Liu - School of Economics and Management, Southwest University of Science and Technology, Mianyang, 621010, China;

E-Mail:liuliang@swust.edu.cn

**List of the supporting information:**

**Supplementary Table S1.** Government governance level indicator system

**Supplementary Table S2.** Robustness tests for replacement policy implementation year and dynamic time window

**Supplementary Table S3.** Comparison of sample means before and after matching

**Supplementary Table S4.** Carbon emissions trading system and energy efficiency: PSM-DID model

**Supplementary Table S5.** Carbon emissions trading system and energy efficiency: controlling for the impact of other energy policy

**Supplementary Table S6.** Calculation results of energy carbon emission rebound effect

**Supplementary Table S1.** Government governance level indicator system

| First level indicator | Second level indicator |  |
| --- | --- | --- |
| Business Environment | Provincial Business Environment Index |  |
| Private economic development level | Percentage of privately listed companies | Number of private listed enterprises / Number of national listed enterprises |
| Innovation Environment | The proportion of R&D investment in the whole of society | Total social R&D investment/GDP |

**Supplementary Table S2.** Robustness tests for replacement policy implementation year and dynamic time window

| Dependent variables | Replacement policy implementation year | Dynamic time window test | | | |
| --- | --- | --- | --- | --- | --- |
|  |  | 1 year | 2 year | 3 year | 4 year |
| SFE | 0.182^***^ | 0.064^**^ | 0.085^***^ | 0.110^***^ | 0.122^***^ |
|  | （-4.54） | （2.14） | （3.30） | （4.47） | （5.16） |
| TFE | 0.084^**^ | 0.076 | 0.0817 | 0.099^**^ | 0.112^***^ |
|  | （-2.59） | （0.74） | （1.37） | （2.23） | （3.11） |
| Sample size | 420 | 90 | 150 | 210 | 270 |

**Supplementary Table S3.** Comparison of sample means before and after matching

| Variables | Unmatched Matched | Mean | | %bias | %reduct | t-test | | v(T)/V(C） |
| --- | --- | --- | --- | --- | --- | --- | --- | --- |
|  |  | Treated | Control |  | \|bias\| | t | p>\|t\| |  |
| industry | U | 0.3757 | 0.4114 | -38.2 | 72.7 | -3.34 | 0.001 | 1.51 |
|  | M | 0.4242 | 0.4340 | -10.4 |  | -0.74 | 0.460 | 1.10 |
| density | U | 7.7858 | 7.8780 | -22.0 | 34.1 | -1.74 | 0.083 | 0.76 |
|  | M | 7.8170 | 7.7563 | 14.5 |  | 1.04 | 0.300 | 0.61 |
| urban | U | 0.7309 | 0.5221 | 174.4 | 91.7 | 16.26 | 0.000 | 2.21^*^ |
|  | M | 0.6511 | 0.6337 | 14.5 |  | 0.90 | 0.370 | 1.70 |
| structure | U | 1.5286 | 0.7671 | 94.8 | 94.8 | 11.68 | 0.000 | 25.53^*^ |
|  | M | 0.8828 | 0.9225 | -4.9 |  | -0.95 | 0.343 | 1.10 |

**Supplementary Table S4.** Carbon emissions trading system and energy efficiency: PSM-DID model

| Variables | SFE | TFE |
| --- | --- | --- |
| treat×period | 0.1403^***^ | 0.0938^**^ |
|  | (3.68) | (2.39) |
| Cons_ | 8.8596^***^ | 3.7875^***^ |
|  | (12.63) | (7.32) |
| Controls | Yes | Yes |
| Year | Yes | Yes |
| Province | Yes | Yes |
| N | 390 | 390 |
| Adj-R^2^ | 0.9843 | 0.8662 |

**Supplementary Table S5.** Carbon emissions trading system and energy efficiency: controlling for the impact of other energy policy

| Variables | SFE | TFE |
| --- | --- | --- |
| treat×period | 0.1040^***^ | 0.0868^**^ |
|  | (2.88) | (2.52) |
| Cons_ | 8.2127^***^ | 3.7020^***^ |
|  | (9.94) | (7.89) |
| Controls | Yes | Yes |
| Year | Yes | Yes |
| Province | Yes | Yes |
| N | 420 | 420 |
| Adj-R^2^ | 0.9784 | 0.9115 |

**Supplementary Table S6.** Calculation results of energy carbon emission rebound effect

| Year | RECE | Year | RECE |
| --- | --- | --- | --- |
| 2007 | -1.808 | 2014 | 0.291 |
| 2008 | -0.280 | 2015 | 0.199 |
| 2009 | -0.728 | 2016 | 0.275 |
| 2010 | -0.036 | 2017 | 0.491 |
| 2011 | 0.108 | 2018 | -0.351 |
| 2012 | 0.254 | 2019 | -0.823 |
| 2013 | 0.197 | 2020 | 0.206 |
